# Development and Implementation of Image-based Algorithms for Measurement of Deformations in Material Testing

**DOI:** 10.3390/s100807469

**Published:** 2010-08-10

**Authors:** Luigi Barazzetti, Marco Scaioni

**Affiliations:** Department of Building Engineering Science and Technology, Politecnico di Milano, *via* Marco d’Oggiono, 18/a, Lecco, Italy; E-Mail: luigi.barazzetti@polimi.it

**Keywords:** automation, computer vision, construction materials, displacement/deformation, image metrology, photogrammetry, vision metrology, targets

## Abstract

This paper presents the development and implementation of three image-based methods used to detect and measure the displacements of a vast number of points in the case of laboratory testing on construction materials. Starting from the needs of structural engineers, three *ad hoc* tools for crack measurement in fibre-reinforced specimens and 2D or 3D deformation analysis through digital images were implemented and tested. These tools make use of advanced image processing algorithms and can integrate or even substitute some traditional sensors employed today in most laboratories. In addition, the automation provided by the implemented software, the limited cost of the instruments and the possibility to operate with an indefinite number of points offer new and more extensive analysis in the field of material testing. Several comparisons with other traditional sensors widely adopted inside most laboratories were carried out in order to demonstrate the accuracy of the implemented software. Implementation details, simulations and real applications are reported and discussed in this paper.

## 1. Introduction

Deformation measurement during laboratory testing on construction materials aims at determining the intrinsic characteristics of the considered object. The examination of the deformation and the knowledge of the applied load (e.g., a mechanical or thermal load) allows the analysis of the mathematical model that describes the behaviour of a construction element.

Several instruments can be used to measure object deformations during loading tests. However, the most widely adopted tools are *linear-variable-differential-transformers* (LVDTs) and *strain gauges* [1], which provide the magnitude of the displacement with the investigation of the changes of electrical resistance due to a load. These tools are considered proven techniques, with an accuracy of ±1 μm or even less, and they give real-time data. On the other hand, they only provide 1D measurements limited to the area in which the sensor is fixed. In addition, a connection with a control unit is necessary and after destructive tests these kinds of sensors can be damaged. Thus, LVDTs or strain gauges are not a convenient choice in the case of extensive analysis on the whole body, in which a great number of 3D points with a good spatial distribution must be measured.

Image-based methods can analyse the whole deformation field of a body by tracking a vast number of points distributed on the object. Images contain all the information to derive 3D measurements from multiple 2D image coordinates with limited cost and good accuracies. In fact, image-based techniques have been used in several applications which involve the determination of the shape of a body and its changes, with satisfactory results in terms of completeness, precision and time [2–5]. These are also known as vision metrology applications. Some commercial cameras (or photogrammetric ones), tripods, light sources and synchronization devices are the components needed to obtain high precision 3D measurements for a large number of points. However, the extraction of 3D information from 2D images is not a simple issue and algorithms for image processing must be developed in order to obtain an automated elaboration.

The goal of image-based methods in material testing is the estimation of accurate 3D coordinates starting from 2D measurements in the images through a perspective mathematical formulation between the object and its projection into several images. Some commercial software allow the analysis of the dynamic changes of several targets distributed on the object in an fully automatic way, but if markerless images are employed no commercial automatic solutions are available on the market. Moreover, the procedure becomes a full-field non-contact technique only without targets, when the natural texture of the object is directly used (generally after a preliminary enhancement with filters that modify the local contrast of the image). For instance, this kind of analysis provides the detection and the measurement in fluids, where LVDTs and strain gauges cannot be employed.

Basically, the precision achievable with image-based techniques depends on the size of the investigated elements [6]. For experiments in a controlled environment a standard deviation of the object coordinates in the order of 1:100,000 of the largest object dimension is expected, but during analysis in repeatable system configurations (e.g., with fixed cameras) a precision of 1:250,000 has been achieved [7,8]. In [9] a hyper redundancy network is used for the study of the deformations of a radio telescope, with an accuracy in the range of 1:580,000 to 1:670,000 obtainable through the use of more images than those strictly necessary. For instance, two images per station enhance the effective angular measurement resolution of a factor of 1.4, while four images per station lead to a factor of 2. In film-based photogrammetric measurements of big antennas this idea has led to an accuracy approaching one part in a million [10].

As the technological development of commercial low-cost cameras is rapidly increasing, image-based methods and low-cost software are commonly used in several sectors (e.g., archaeology [11], geology [12], medicine [13]) with good results in terms of precision. However, photogrammetric methods have a limited use for material testing in civil engineering. This is mainly due to the lack of automatic processing algorithms and user-friendly software, especially in the case of markerless images.

Some low-cost digital cameras and targets can be a convenient solution for the analysis of the whole surface of an object. The employed targets can be really inexpensive (a piece of white paper with a black mark is sufficient for many applications), while in the case of more exhaustive experiments they can be printed on metal plates or can be made of retro-reflective materials. The centre of the target can be automatically measured with a high precision (up to ±0.01 pixel) in a fully automated way, improving the precision of the corresponding 3D coordinates.

A group of targets permanently fixed on the object provides a regular mesh for all deformation analyses. These dense points can approximate the deformation field of the whole body. A fundamental advantage of an image-based method is the possibility of analysing more targets than those strictly necessary, without increasing the cost of the test and with a limited worsening of the processing time. However, in some applications targets cannot be employed (e.g., for fluid elements) and automatic methods based on the natural texture of the body must be developed. This kind of analysis is more complicated, especially in the case of bad surfaces without details. This fact limits the use of image-based methods inside civil engineering laboratories.

This paper presents three image-based algorithms capable of analysing the deformation field of a generic object during a loading test. These methods work with targets but also with markerless images and can determine the 3D coordinates of a huge number of points in an automatic way. They are currently employed in some civil engineering laboratories, where several building materials and structural elements are tested with satisfactory results in terms of accuracy. In several applications these methods integrate or substitute traditional sensors and provide additional information, which are useful for more complete and detailed investigations.

We focus on the measurement of a finite number of points with a good distribution, while other existing approaches present the extension of the measurement problem to the whole surface of the body [14]. Our choice is motivated by the needs of structural engineers, who were interested in the analysis of particular points in crucial locations. Another different approach is presented in [15], in which a system for modelling the interaction behaviour of real objects (including deformations) was developed. A fully automated image-based measurement system is described in [16].

The first tool here presented allows the estimation of crack variations in fluid fibre-reinforced specimens (Section 2). This is a new non-conventional application for which there are no commercial solutions. This task required the development of an *ad hoc* sensor for image acquisition and an algorithm for the automatic identification of the cracking process. In particular, a CMOS sensor was transformed into a crackmeter device. The other tools were developed for dynamic 2D and 3D measurements on standard structural elements (e.g., beams, pillars, foundations, walls…) and can operate with targets (like commercial software) but also without markers (Section 3). This last option provides new measurements during tests where only limited information can be achieved with traditional sensors. In order to demonstrate the potential of these photogrammetric methods, a theoretical explanation and several examples are shown and discussed, with a check of the achievable accuracy. Moreover, the advantages (and disadvantages) about the use of low-cost digital cameras are reported and discussed, with a comparison with traditional sensors.

## 2. Crack Aperture Estimation in Fluid Specimens

### 2.1. Overview

Cracks are expected for several construction materials during their service ability [17], especially in the case of reinforced-concrete elements. However, a significant variation of the crack aperture can lead to a progressive deterioration of the steel reinforcement rod with a consequent worsening of the stability of the structure. For these reasons, crack monitoring plays a fundamental role during inspections and laboratory probes. The study of new techniques able to avoid or mitigate the cracking process is a field of research of primary importance in civil engineering. Some innovative solutions based on the use of fibres (in addition to existing mixtures) demonstrated the possibility to limit the propagation of the cracks.

Laboratory testing on fibre-reinforced specimens allow one to study the effect of different fibres and mixture components (water, cement, sand…), in order to determine the best compromise for real applications. A traditional analysis is based on the study of the aperture, shape, location and orientation of cracks with small specimens that simulate the behaviour of the real object.

To monitor the aperture of a crack during standard tests strain gauges are generally used. However, civil engineers needed more exhaustive and specific measurements than those achievable with these standard sensors. In fact, strain gauges allow only one-point and one-dimensional measurements [18], after a stable application of the sensor on the specimen. These kinds of information are useful in the case of traditional laboratory applications, but they are insufficient for exhaustive and detailed scientific analysis focused on the development of innovative materials. Indeed, the needs of civil engineers required the development of more advanced solutions.

Another issue regards the state of the body: all measurements on the specimens begin after the casting, when the specimens is liquid, and preliminary data about the number of expected cracks and their positions are not available. For these reasons, a new solution capable of analysing the deformations in these particular working conditions was necessary.

**Figure 1.**
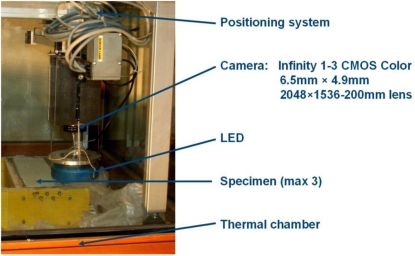
The developed system for crack aperture estimation.

The developed tool for such measurements is composed of a mechanical arm carrying a digital camera (Figure 1) and an algorithm for automatic multi-image processing. A thermal chamber, which contains the sensor used to photograph the specimens, allows controlled and stable testing conditions. Then, an automated positioning system moves a CMOS INFINITY 1–3 camera (3.1 megapixels) equipped with a 200 mm lens over the specimen. All images are captured for several positions at different epochs by planning the trajectory of the mechanical arm and the number of shots. Each image covers an area of 18 mm × 13.8 mm roughly and its inspection allows the detection of very small details. The robotic arm moves the camera along prefixed directions in order to photograph the whole specimens. This operation can be repeated several times and the images can be used for a multi-epoch investigation to detect dynamic variations. As a typical test may require several days, hundreds of images can be collected. Therefore, a useful choice (before analysing all images) is related to a rapid visual check of the images of the last epoch in order to select the images which contain a crack. The remaining images can be removed from the elaboration to speed up the whole processing.

### 2.2. Crack detection and Image Coordinate Measurement

The estimation of the crack aperture is carried out with an automated algorithm capable of detecting a crack in each single image by measuring its border coordinates (in pixels). Then, a procedure based on simple geometric considerations between the camera and the specimen allows the estimation of the crack aperture in metric units.

Image coordinates can be automatically measured with the methodology proposed in [19], in which a tool named IMCA (IMage Crack Aperture) was developed by the authors for crack measurement during structure inspections. The same procedure is used in these experiments, but some changes were necessary to adapt the algorithm in the case of fibre-reinforced specimens and repetitive experimental conditions. The new procedure uses a filtering algorithm that detects the image coordinates of a crack by means of an intelligent reduction of the colour depth. This technique can be assumed as a conversion of the original RGB image to a new binary image (0 is the crack while 1 means background). As the radiometric content of a generic image is expressed by three functions which correspond to the colours (red, green and blue) of Bayer’s filter [20] covering the sensor, this new strategy uses this information to automate the measurement phase. We denote these functions as *R*(*i*, *j*), *G*(*i*, *j*) and *B*(*i*, *j*), in which (*i*, *j*) are the coordinated of a generic pixel. The behaviour of the RGB functions along a cross-section of a crack is quite simple: they rapidly decrease and increase in the crack and have a quite constant value far from the crack. Starting from these simple considerations, a filtering algorithm was developed. It is based on the minimum values *R*_min_, *G*_min_, *B*_min_ of the functions (in the middle of the crack) and the values *R*_A_ *G*_B_ *B*_C_ in which the slope of the functions changes. These values can be estimated with the analysis of some cross-section and then used for the completion of the test. A “global level” *L* can be estimated for the crack as follows:

(1) $$L=\frac{1}{3}\left[\frac{R_{\text{min}}}{R_{A}}+\frac{G_{\text{min}}}{G_{B}}+\frac{B_{\text{min}}}{B_{C}}\right]$$

and represents the parameter to filter the image. For any generic pixel (*i*, *j*) of the image a local level *L’*(*i*, *j*) can be estimated by considering its radiometric content:

(2) $$L\prime (i,j)=\frac{1}{3}\left[\frac{R_{\text{min}}}{R_{P}}+\frac{G_{\text{min}}}{G_{P}}+\frac{B_{\text{min}}}{B_{P}}\right]$$

The creation of the filtered image is carried out by comparing *L’* and *L* as follows:

**Table:** 

*L’*(*i*, *j*) − *L* > 0	pixel ∈ CRACK;
*L’*(*i*, *j*) − *L* = 0	pixel ∈ BORDER;
*L’*(*i*, *j*) − *L* < 0	pixel ∉ {CRACK or BORDER}.

This method uses the whole RGB content of an image, while other existing techniques (e.g., [21,22]) work with their combinations and need a preliminary conversion to create a new gray-scale image. This choice is motivated by the results obtained with the proposed methodology, although we are testing a procedure which uses the green channel.

The main advantage during laboratory testing, with repetitive working conditions, is the possibility of estimating a preliminary global level, which can be considered a constant for specific applications. In fact, if illumination conditions are stable (in this case a LED is permanently employed for all images) the global level does not vary significantly during the test. In addition, small errors in this phase can be considered systematic errors and can be removed during the estimation of the aperture variations. After some tests we estimated an optimal level for fibre-reinforced concrete elements equal to 0.19.

### 2.3. Crack Aperture Estimation

To estimate the crack aperture a transformation between image and object spaces must be employed. As the analysis starts with a fluid specimen (its external surface is horizontal), the robotic arm was assembled in order to generate 2D horizontal movements. With this particular configuration, image and object planes (or camera sensor and specimen surface) are parallel and the camera maps the object through a similarity transformation where the scale is the only unknown. This simple solution allows an easy computation of object coordinates without using more complex transformations requiring the knowledge of several parameters. A more detailed description about this procedure is shown in Section 3.2, because an extension of this transformation is used in another tool, while for this particular application the scale number is the ambiguity.

**Figure 2.**
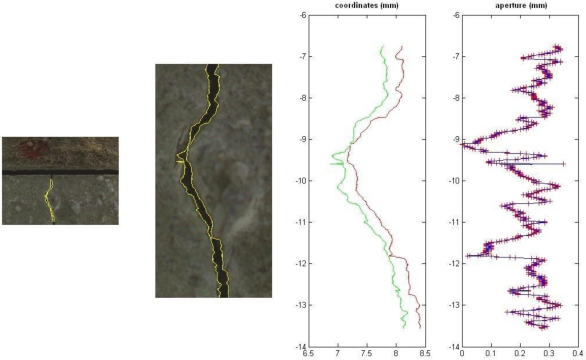
Some results with the implemented software: crack borders and the estimated aperture.

The scale factor was estimated by measuring the size of a pixel projected onto a reference object (a small metal plate) placed on the specimen. The size of this object was measured with a calliper: it is sufficient to divide the width of the plate by the number of pixel picturing the object to determine the scale factor. With the INFINITY camera a pixel covers an area of 9 μm × 9 μm, which is also the accuracy of the implemented tool (see next section for further details). The output interface of the tool, which gives a graphical and numerical visualization of the crack aperture, is shown in Figure 2. The procedure is quite simple: the user just has to select a crack in an image and the dynamic analysis can be carried out in automatic way. This task is performed by considering corresponding images taken at different epochs. Lastly, the aperture variations can be estimated by using the variations detected at different epochs (relative measurements).

### 2.4. Accuracy of the Method

**Figure 3.**
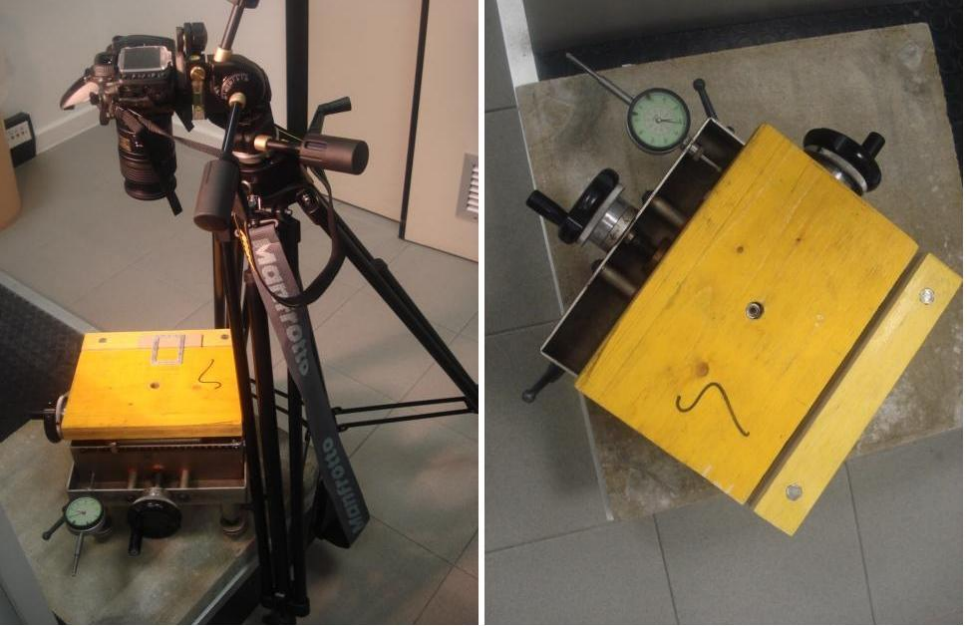
The sledge used to check the accuracy of the image-based tool.

To check the accuracy of the implemented method a comparison with other sensors is mandatory. Nowadays, a system capable of measuring the aperture variations in fluid elements with an accuracy and a density better than the implemented tool is not available. This means that accuracy cannot be checked with experiments on fluid specimens. To overcome this drawback we developed an alternative solution with a solid object and a special micrometric sledge (Figure 3), which is composed of two plates (the first one is fixed while the second one can be moved with two micrometric screws in order to simulate a planar motion). Two mechanical gauges provide the magnitude of the displacements with an accuracy superior to ±0.01 mm. The sledge allows one to simulate the aperture of a “synthetic crack”, where all points have the same displacement (rigid motion). Anyway, this is sufficient to check the accuracy of the image-based method.

A Nikon D80 camera equipped with a 90 mm lens was placed over the sledge in order to determine the simulated variation with the implemented tool. The mathematical relation between image and object spaces was estimated with a special calibration frame, composed of points with known coordinates (see Section 3.2). From a theoretical point of view, the precision of object coordinates *σ_XY_* can be estimated with a simple formula:

(3) $$\sigma_{\mathrm{XY}}=\pm \frac{d}{c}\sigma_{\mathrm{xy}}$$

where *d* is the camera-object distance, *c* the focal length of the camera and *σ_xy_* the image coordinate precision. The fundamental assumption of Equation 3 is the parallelism between image and object planes.

However, Equation 3 gives a theoretical precision that must be compared with real data (useful for a preliminary knowledge about the expected accuracy). In our tests we placed the camera with a distance *d*_1_ equal to 600 mm, then we reduced the distance to *d*_2_ = 220 mm. Both mechanical and image-based measurements were compared and the results showed a standard deviation of the differences of ±0.037 mm (*d*_1_ = 600 mm) and ±0.012 mm (*d*_2_ = 220 mm). Supposing that the precision of the filtering algorithm is equal to ±1 pixel, a theoretical precision of ±0.04 mm and ±0.014 mm can be estimated with the camera used in both configurations (pixel size is 0.0061 mm). This means that the precision of the implemented tool is equal to the GSD (Ground Sampling Distance), which represents the projection of a pixel onto the object. To improve the precision of the object coordinates the camera-object distance can be reduced or the focal length can be increased. However, in both cases the angle of view is progressively reduced and a smaller part of the object can be imaged. The best choice is a compromise between precision and imaged area. Several other comparisons validated the proposed results and confirm the expected accuracy in the case of the INFINITY camera (a pixel projected onto the object is ±0.009 mm).

## 3. 2D Deformation Measurements

### 3.1. Overview of the Implemented Method

During some tests the analysis of 3D movements is not strictly necessary. In fact, if the analyzed object is flat (e.g., the external surface of a beam), the estimation of a 2D motion is more than sufficient for several experiments. This fact leads to a simplification of the measurement problem, with a reduction of the degrees of freedom for a generic point of the object. Moreover, there is an advantage in terms of cost: a single image for each epoch becomes sufficient to analyze the movements of all points. Starting from the image coordinates of point (*x_i_*, *y_i_*) the corresponding object coordinates (*X_i_*, *Y_i_*) can be calculated by using a 2D homography. Beyond the reduction of the number of cameras (a fixed camera is sufficient, thus synchronization devices are not mandatory), a rigorous calibration of the camera is not needed. However, the influence of an uncalibrated camera on the final results should be carefully considered, although the whole operation can be carried out without knowing the intrinsic parameters of the camera used. This could be an advantage when no information about the used sensor is available. This aspect is analyzed with more details in Section 3.5.

The equipment includes a camera placed on a tripod and an algorithm able to track all image points and to estimate real movements. The acquisition frequency depends on several factors and varies with the investigated object and the selected load. For this reason it is not possible to fix an optimal value for every experiment. This means that an *ad-hoc* sampling frequency must be estimated before the beginning of the test by considering several factors (e.g., load, expected deformation, object, texture…). Probably, the best solution is to acquire more images than those strictly needed. Then, images can be decimated.

The implementation of an *ad-hoc* software was necessary because commercial solutions for fully automated image processing are not available on the market. Some commercial packages (e.g., Australis, iWitness, PhotoModeler…) work with targets, but if markers cannot be employed the elaboration needs tedious interactive measurements. In addition, these software packages generally work with two or more cameras, while the situation with a single image needs a different mathematical formulation.

### 3.2. Target Localization and Matching

Several targets distributed on the object are a valid support in image-based deformation measurements. A regular mesh of targets allows one to analyze the whole surface of the body, while the use of traditional sensors (e.g., strain gauges or LVDTs) increases the cost and needs complex connections with control units. For these reasons, photogrammetric targets are a cheap solution with a simple connection on the analyzed body. During several real surveys, all targets can be printed (e.g., a black dot with a white background), while for more advanced and extensive analysis they can be made of metal.

**Figure 4.**
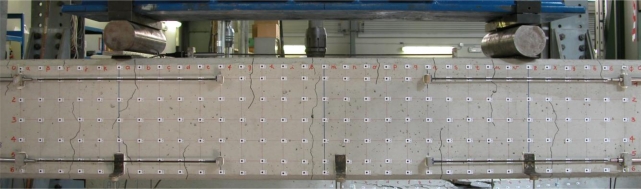
A beam can be considered a flat object.

Figure 4 shows a typical analysis with targets: the surface of the beam can be considered a flat object and a regular mesh allows the measurement of its deformation field. In this case, six LVDTs were used, but they offer few measurements along prefixed directions. With this in mind, the target-based image solution is more convenient.

All targets can be automatically matched by using a 2D normalized cross-correlation technique between a target template and the image [23]. Basically, the method supposes that the target template (a perfect image of the target, which can be easily created with any software for image visualization or editing) is similar to the target used during the survey.

An automated search of the target(s) in the whole image can be carried out by comparing the template with the local content of the image (a preliminary conversion of the original RGB image to a new grayscale one must be performed). The measurement of the centre of the target in the image is carried out by moving the template *f*(*x*, *y*) in a search window (or in the whole image), with a sequence of small displacements (e.g., one or two pixels). For each position the normalized correlation coefficient *ρ*(*x*, *y*) between *f*(*x*, *y*) and the local content of the image *g*(*x*, *y*) (“patch”) can be estimated with the relation:

(4) $$\rho (x,y)=\frac{\sum_{u=-N}^{N}\sum_{v=-M}^{M}\left(f(x\prime +u,y\prime +v)-\mu_{f}\right)\cdot \left(g(x+u,y+v)-\mu_{g}\right)}{{\left(\sum_{u=-N}^{N}\sum_{v=-M}^{M}{\left(f(x\prime +u,y\prime +v)-\mu_{f}\right)}^{2}\sum_{u=-N}^{N}\sum_{v=-M}^{M}{\left(g(x+u,y+v)-\mu_{g}\right)}^{2}\right)}^{1/2}}$$

where (*x*, *y*, *x’*, *y’*) are the centers of template and patch and *μ_f_* and *μ_g_* are the mean values of intensity of *f* and *g*. The size of the patch is (2*N* + 1) × (2*M* + 1) pixels. The centre of the target can be assumed at max [*ρ*(*x*, *y*)] with an additional constraint on the minimal value (e.g., 0.7). Sub-pixel precision can be achieved by estimating the first derivative of *ρ*(*x*, *y*) [24].

This method is easy to implement and fast from a computational point of view [25], but it takes into account only two shifts between template and image. In the case of real surveys there are several other deformities such as scale variations or rotations, affine deformations, illumination changes and so on. They lead to poor results with this basic geometric model. In [26] a modified cross-correlation approach was developed to consider all these deformities by reshaping the patch with an affine transformation, which is more suitable for real surveys. However, we prefer to use the method proposed in [27] and coined *Least Squares Matching* (LSM).

Starting from a perfect similarity between the template and the patch:

(5) f(x,y)=g(x,y)

the LSM method takes into account a more realistic situation, in which equation 5 is not consistent and a noise *e*(*x*, *y*) is added:

(6) f(x,y)−e(x,y)=g(x,y)

To operate with the Gauss-Markov Least Squares estimation model *g*(*x*, *y*) must be linearized at an approximate location with a first order Taylor’s expansion:

(7) $$f(x,y)-e(x,y)=g(x_{0},y_{0})+{\left(\frac{\partial g(x_{0},y_{0})}{\partial x}\right)}_{0}\mathrm{dx}+{\left(\frac{\partial g(x_{0},y_{0})}{\partial y}\right)}_{0}\mathrm{dy}=g^{0}+g_{x}^{0}\mathrm{dx}+g_{y}^{0}\mathrm{dy}$$

where an affine transformation is considered as geometric model (a radiometric correction is not used because illumination conditions are stable if light sources are used during experiments in controlled conditions):

(8) $$\begin{array}{l} x=a_{0}+a_{1}x_{0}+a_{2}y_{0}\\ y=b_{0}+b_{1}x_{0}+b_{2}y_{0} \end{array}$$

The parameters *a*_0_ and *b*_0_ (shifts) are unknown values that indicates the centre of the target, while the other coefficients can be used to adjust shape deformations. Equation 6 can be cast in the form:

(9) $$\begin{array}{l} f(x,y)-e(x,y)=g^{0}(x,y)+g_{x}{\mathrm{da}}_{0}+g_{x}x_{0}{\mathrm{da}}_{1}+\\ +g_{x}y_{0}{\mathrm{da}}_{2}+g_{y}{\mathrm{db}}_{0}+g_{y}x_{0}{\mathrm{db}}_{1}+g_{y}y_{0}{\mathrm{db}}_{2} \end{array}$$

Finally, the unknown parameters can be grouped into a vector:

(10) $$x={\left[{\mathrm{da}}_{0},{\mathrm{da}}_{1},{\mathrm{da}}_{2},{\mathrm{db}}_{0},{\mathrm{db}}_{1},{\mathrm{db}}_{2}\right]}^{T}$$

and the system can be written as:

(11) Ax=1−e

in which *l_k_* = *f*(x, y) − *g* (*x*_0_, *y*_0_). The solution is given by:

(12) $$x={\left(A^{T}A\right)}^{-1}A^{T}1$$

In order to complete the linearization with a Taylor’s expansion, a set of initial approximations for the unknowns is chosen as follows:

(13) $${\mathrm{da}}_{0}={\mathrm{db}}_{0}=0\;\;;\;\;{\mathrm{da}}_{1}={\mathrm{db}}_{2}=1\;\;;\;\;{\mathrm{da}}_{2}={\mathrm{db}}_{1}=0$$

and then the solution is computed iteratively with a stop criteria (e.g., on the estimated sigma-naught). In the implemented version of the LSM algorithm, some tests to check the determinability of parameters (10) were included; further information about this aspect can be found in [28].

The LSM method ensures high precision measurements (up to ±0.01 pixels) and is an optimal choice in the case of targets. However, it cannot be considered as an alternative to cross-correlation: cross-correlation provides good approximate values about target locations and LSM refines center coordinates. Thus, the combined use of both these techniques is strictly mandatory in order to automate the whole analysis.

### 3.3. Computation of Object Coordinates and Dynamical Analysis

Object coordinates can be calculated by using image coordinates and a transformation between image and object spaces. In the case of flat objects all points lie on the same plane and the mathematical transformation between image and object spaces can be described with a 2D homography.

The relation between an image point in homogenous coordinates (*x_i_*, *y_i_*, 1)*^T^* and the corresponding object coordinates (*X_i_*, *Y_i_*, 1)*^T^* is:

(14) $$\left[\begin{array}{c} X_{i}\\ Y_{i}\\ 1 \end{array}\right]=\left[\begin{array}{ccc} a_{1} & a_{2} & a_{3}\\ b_{1} & b_{2} & b_{3}\\ c_{1} & c_{2} & c_{3} \end{array}\right]\left[\begin{array}{c} x_{i}\\ y_{i}\\ 1 \end{array}\right]$$

or, in a compact form:

(15) $$X_{i}={\text{Hx}}_{i}$$

**H** contains the parameters of the transformation and has a rank deficiency, thus only eight elements are independent and an external constraint must be used (we set the last elements *c*_3_ equal to 1). To obtain inhomogeneous coordinates it is sufficient to divide image and object coordinates by their third coordinate.

This leads to the inhomogeneous form of the planar homography:

(16) $$\frac{X_{i}}{1}=\frac{a_{1}x_{i}+a_{2}y_{i}+a_{3}}{c_{1}x_{i}+c_{2}y_{i}+1}\;\;\;\;\;\;\;\;\;\frac{Y_{i}}{1}=\frac{b_{1}x_{i}+b_{2}y_{i}+b_{3}}{c_{1}x_{i}+c_{2}y_{i}+1}$$

which are linear in the elements of **H**. In fact, a multiplication by the denominator leads to:

(17) $$\begin{array}{l} c_{1}x_{i}X_{i}+c_{2}y_{i}X_{i}+X_{i}-a_{1}x_{i}-a_{2}y_{i}-a_{3}=0\\ c_{1}x_{i}Y_{i}+c_{2}y_{i}Y_{i}+Y_{i}-b_{1}x_{i}-b_{2}y_{i}-b_{3}=0 \end{array}$$

To estimate the eight coefficients of **H** some correspondences between the image and object spaces must be known (at least four points). Given *m* corresponding points, Equations (17) provide a system of 2*m* equations that can be solved *via* Least Squares [29].

The measurements of the object points needed to estimate **H** can be carried out with several techniques (e.g., total station, calibrated frames …). Moreover, **H** is estimated for the first epoch and then assumed constant during the next phases. Indeed, if the camera is placed on a stable tripod the transformation does not change and 2D displacements can be directly estimated by using image coordinates.

A better strategy to visualize the results is based on the removal of the perspective effect from the images. Here, the homography **H** estimated for the first image can be applied to all images before measuring the image coordinates. The measurement of image coordinates with the rectified images provides object coordinates.

**Figure 5.**
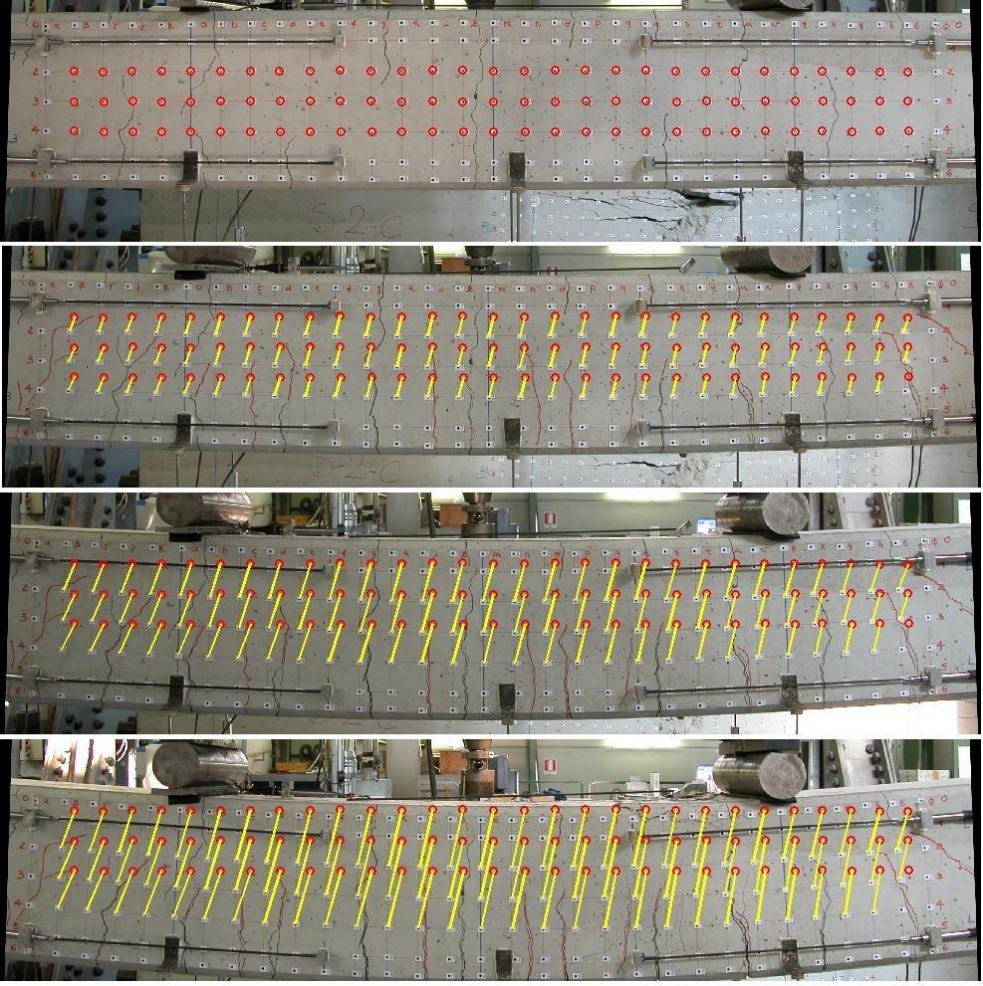
Some rectified images of the sequence and the magnitude of the displacements.

Figure 5 shows some rectified images for the beam sequence. As can be seen, images present a distortion that cannot be removed if calibration is unknown. This generates an error in the final results (more details about the influence of image distortion are presented in Section 3.5). The circle represents the position of each target for the first epoch, while the length of each line gives 2D movements (a known scale factor is needed).

**Figure 6.**
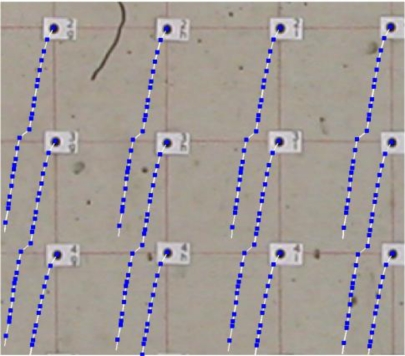
Target displacements projected onto the initial rectified image.

Figure 6 shows a detail. In this case the positions of some targets measured at different epochs are shown, in which the first image is used for this visualization. Target coordinates can be measured interactively and this kind of visualization offers a global visualization about the deformation field.

### 3.4. Automated Elaboration of Target-Less Images

Targets are very useful to monitor deformations emerging in loading tests: they can be easily measured with a high precision and their application onto the body is simple and cheap. However, in some cases targets cannot be permanently installed or can be lost during the test. To overcome this drawback a synthetic texture can be generated (e.g., by painting the object) but we developed a new solution capable of working with target-less images. It uses the natural texture of the object after a preliminary image enhancement. Interest operators can be used to detect a sufficient number of features in the first image of the sequence. Then, these features are tracked with the proposed methodology based on cross-correlation and LSM along the sequence.

Before the beginning of the test it is highly recommended to process some images. This operation is really useful to verify the quality of the images and the possibility to use the natural texture (i). In the case of a failure with the natural features, a procedure based on synthetic corners (ii) can be used. The application of targets onto the object remains the last choice when the previous method cannot be employed.

Several features can be detected in an image (e.g., corners, edges, regions…) and several operators are available (probably too many to be listed here). For a more exhaustive review the reader is referred to [30–32]. The method used in the implemented tool is the FAST (Features from Accelerated Segment Test) operator [33], which is a corner detector for high speed processing, today employed in video analysis and tracking. The functioning of FAST is based on the analysis of a circle of 16 pixels around a generic corner *p*. A pixel is a corner if *n* contiguous pixels are all brighter than the intensity *I_p_* of the candidate pixel plus a threshold *t*, or all darker than *I_p_* − *t*.

The choice of this operator is supported by the impressive number of corners that can be extracted from an image. However, corners are extracted only for the first image of the sequence, while for the next ones a tracking *via* cross-correlation and LSM is used.

In some cases images might present a bad texture and a limited number of corners could be extracted. In addition, the distribution of points could be inhomogeneous. To solve this problem a procedure based on a preliminary image enhancement can be used. Many methods are today available and generally work by considering global parameters: most software for image enhancement have automatic functions capable of modifying the contrast of the image, but the same level is used for the whole image. If a homogenous distribution of all points is needed this can lead to a poor solution. This is the reason why we prefer to optimize the contrast locally. Wallis [34] proposed an *ad hoc* image filter which splits the image into small rectangular blocks. These blocks are progressively analyzed by considering their local statistics.

The Wallis filter has the form:

(18) $$I_{f}(x,y)=I_{o}(x,y)r_{1}+r_{0}$$

where:

(19) $$r_{1}={\mathrm{cs}}_{o}\left({\mathrm{cs}}_{o}+\frac{s_{t}}{c}\right)$$

and:

(20) $$r_{0}={\mathrm{bm}}_{t}+(1-b-r_{1})m_{o}$$

*I_f_* and *I_o_* are the filtered and original images, *r*_0_ and *r*_1_ the additive and multiplicative parameters, *m*_o_ and *s*_o_ the mean and standard deviation of original images, *m_t_* and *s*_t_ the target mean and standard deviation for the filtered images, *c* the contrast expansion constant and *b* the brightness forcing constant. Basically, the user has to select the block size: a small block (e.g., 7 × 7 pixels) results in a strong enhancement, while a large block (e.g., 131 × 131 pixels) generates a loss of detail.

For each single block *m*_o_ and *s*_o_ are estimated and the resulting values are assigned to the central pixel of each block, while for other pixels these values are estimated with a bilinear interpolation. The target mean and standard deviation *m_t_* and *s_t_* are manually selected. Normally, for an 8-bit image a good choice of the target mean is 127, while the target standard deviation value can be 50. Good values for the constant expansion constant *c* are in the range [0.7, 1], while for the brightness forcing constant *b* the suggested range is [0.5, 1]. An optimal combination of all these parameters can be determined with few tries in which a visual check of the filtered image is sufficient. Moreover, it is possible to extract FAST corners and check their number and distribution.

**Figure 7.**
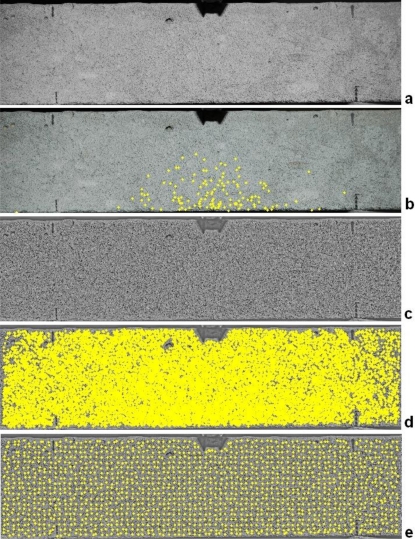
Results in the case of markerless image sequences: (a) original image and (b) extracted corners, (c) filtered image and (d) extracted corners, (e) corner reduction according to a quasi regular grid.

Figure 7 shows the original image of a small beam (a) and the detected corners with the FAST operator (b). As can be seen, few points can be measured. The filtered image (c) provides more corners (d) with a better distribution. At the end of the process points can be mapped (d) onto a “quasi” regular grid (in this case each cell is 50 × 50 pixels roughly).

The dynamic analysis is carried out by filtering all images and tracking the original FAST corners with cross-correlation and LSM along the image sequence, which must be filtered with the same parameters. It is also recommended to use stable illumination conditions during the analysis (e.g., external light sources like lamps), a very high acquisition frequency according to the duration of the test (to limit the differences between consecutive images) and small blocks (e.g., 9 × 9 pixels) for the filtering process (to reduce the effect of local deformations during the test). Moreover, this procedure should be used when limited deformations are expected. With these experimental conditions we verified that only a limited number of points is lost during the sequence analysis.

### 3.5. Influence of Camera Calibration

The mathematical analysis proposed in Section 3.3 demonstrated that no information about the camera used is required when the relation between image and object spaces is a planar homography. Thus, image coordinates and few reference object points are adequate to complete the elaboration. Camera calibration is intended as the process to estimate the intrinsic parameters of the camera, comprehending the principal distance, principal point and distortion coefficients. A good calibration is an essential prerequisite for precise and reliable measurements from images, and is widely adopted in several surveys where high accuracies must be achieved. Several software use an 8-terms model derived from the original formulation for image distortion proposed by Brown [35]:

(21) $$\begin{array}{l} \Delta x=x*(k_{1}r^{2}+k_{2}r^{4}+k_{3}r^{6})+p_{1}(r^{2}+{2x}^{*2})+2p_{2}x*y*\\ \Delta y=y*(k_{1}r^{2}+k_{2}r^{4}+k_{3}r^{6})+p_{2}(r^{2}+{2y}^{*2})+2p_{1}x*y* \end{array}$$

where Δ*x* and Δ*y* are the corrections for a generic image point with coordinates (*x*, *y*), x* = *x* − *x_p_* and y* = *y* − *y_p_* are the image coordinates referred to the principal point, *r*^2^ = x*^2^ − y*^2^ is the squared radial distance.

The coefficients *k*_1_, *k*_2_, *k*_3_ model the radial distortion. In particular, the coefficient *k*_1_ is generally sufficient during most surveys, but when a high accuracy is needed, the coefficients *k*_2_ and *k*_3_ have to be used as well. Tangential distortion, that is due to a misalignment of the camera lenses along the optical axis, can be modeled with *p*_1_ and *p*_2_. The magnitude of tangential distortion is limited if compared to radial distortion, especially with wide-angle lenses.

Digital cameras should be calibrated periodically, because several issues about the stability of the sensor could arise. A standard camera calibration procedure can be performed by using known points (and few images), or without any external information and special coded targets. The former (termed as field calibration) needs external 3D information provided through a framework with several targets, whose 3D coordinates have been previously measured (e.g., with a total station). The latter (self-calibration) is based on a free-net adjustment [36] in which points with known 3D coordinates are not necessary. The calibration framework needs a set of targets, which are measured in the images. Furthermore, some additional mathematical constraints and a block composed of several images with a suitable network geometry are needed. For a general review about calibration methods the reader is referred to [37].

Equations 21 allow one to model image distortion in order to correct each single measured image point. In addition, distortion coefficients can be used to derive distortion-free images, although this operation needs a longer elaboration time. If calibration is given, the correction of image coordinates should be always carried out in order to improve the precision of the final result. However, image distortion can be considered locally constant, thus when different epochs are analyzed its contribute can be assumed as a systematic error and removed by using relative differences. Anyway, this assumption is valid for small point displacements, therefore a camera should be always calibrated especially with consumer cameras and wide-angle lenses.

## 4. 3D Image-based Deformation Measurements

### 4.1. Combining Multiple Images for 3D Analysis

When 3D measurements are necessary at least two images for each epoch are needed. Images must be taken at the same time, thus all cameras must be synchronized. Several images can be used to improve the precision of the object coordinates, however a more expensive instrumentation becomes necessary. Fraser [38] proposed the following formula to estimate the theoretical precision of a 3D image-based survey:

(22) $$\sigma_{\mathrm{XYZ}}=\frac{\mathrm{qS}\sigma_{\mathrm{xy}}}{\sqrt{k}}$$

where *q* is an empirical factor (between 0.4 and 2 according to the number of images and their spatial distribution), *S* is the scale number (camera-object distance divided by the focal length), *σ_xy_* is the precision of image coordinates and *k* is the number of images. Precision can be enhanced by increasing the number of cameras (*i.e.* more observations for the same 3D point), though this improvement is proportional to the square root of the number of images.

The mathematical model for image orientation is based on collinearity equations [35]. An image can be considered as a central projection in space, in which the relationship between an image point (*x_ij_*, *y_ij_*) and the corresponding object point (*X_j_*, *Y_j_*, *Z_j_*) can be written with a 7-parameters transformation:

(23) $$\left[\begin{array}{c} x_{\mathrm{ij}}-x_{\mathrm{pi}}+{\Delta x}_{\mathrm{ij}}\\ y_{\mathrm{ij}}-y_{\mathrm{pi}}+{\Delta y}_{\mathrm{ij}}\\ -c_{i} \end{array}\right]=\lambda_{i}R_{i}\left[\begin{array}{c} X_{j}-X_{0j}\\ Y_{j}-Y_{0j}\\ Z_{j}-Z_{0j} \end{array}\right]$$

for each image *i* and point *j*. In Equation 23 *R_i_* is a rotation matrix, (*X*_0*i*_, *Y*_0*i*_, *Z*_0*i*_) are the coordinates of the perspective centre, *c_i_* is the principal distance, (*x_pi_*, *y_pi_*) are the locations of the principal point in the image *i* and (Δ*x_ij_*, Δ*y_ij_*) are the correction terms for image distortion. Equation 23 is non-linear and a rigorous solution requires their linearization (thus the knowledge of good approximate values) and an iterative approach to estimate the unknowns (*i.e.*, camera orientation parameters and 3D object coordinates). A rigorous bundle solution, coupled with the estimation of the unknown parameters based on the Gauss-Markov model of the Least Squares (LS), provides an efficient, precise and reliable solution in a functional and stochastic sense [39]. The unknown parameters are estimated using proper coefficients to weight the observations with different precisions. Their theoretical precision can be evaluated through the estimated covariance matrix, while the posterior variance of unit weight (*σ*_0_^2^) gives the final quality of the adjustment. The functional model of the system of Equation 23 is solved with the LS solution:

(24) $$x={\left(A^{T}\text{WA}\right)}^{-1}A^{T}W1$$

where **A** is the design matrix, containing the partial derivatives of Equations 23 with respect to the unknowns and evaluated at the approximated values. **W** is a weight matrix, **x** is the unknown vector and **l** is the observation vector. The residuals **v** of the observations and *σ*_0_^2^ can be estimated as:

(25) v=Ax−1

(26) $$\sigma_{0}^{2}=\frac{v^{T}\text{Wv}}{r}$$

where *r* is the redundancy (*i.e.*, the difference between the number of observations and unknowns).

The precision of the estimated unknowns can be retrieved from the covariance matrix:

(27) $$K_{\mathrm{xx}}=\sigma_{0}^{2}{\left(A^{T}\text{WA}\right)}^{-1}$$

The diagonal elements of the **K***_xx_* matrix are the variances of each single unknown, while the other elements represent the covariances between the unknowns.

To invert the semi-definite positive matrix **A***^T^***WA** in Equation 24 an external datum must established. This operation can be performed by fixing seven parameters (three translations, three rotations and a scale factor) and can be carried out in several ways. In our implementation we implemented two strategies:

1. the use of an orientation frame (*i.e.*, a support with known points), which must be placed on the body at the beginning (or at the end) of the test;
2. a free-net adjustment based on inner constraints [40], which requires at least the scale of the project using an element with a known length (e.g., a calibrated bar).

After processing the images of the first epoch with the developed methodology, if cameras are placed on stable supports exterior orientation parameters can be considered constant. Then, the dynamic analysis is based on the measurement of the image coordinates by tracking the points along the image sequences with cross-correlation and LSM. The computation of object coordinates is performed by using the fixed orientation parameters.

An important point is related to occlusions. However, during this kind of analysis the deformation emerging is limited with respect to the size of the object. Therefore a good initial setup of the cameras around the object avoids the creation of occlusions during the test.

### 4.2. Measurement of Image Coordinates

**Figure 8.**
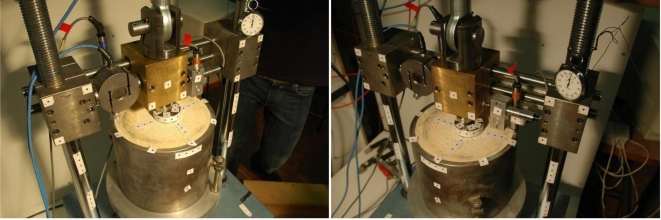
A target-based survey with two synchronized cameras.

In the case of 2D dynamic measurements an image point must be tracked along the image sequence. When multiple views must be analyzed, it is necessary to determine the same point among the images captured at the same epoch, then the point can be tracked along the sequence. The determination of the image correspondences can be carried out by using targets or with the texture of the object. Targets can be automatically detected for all images, but it is often necessary to (manually) select homologous points for the images of the first epoch. However, an opportune coding can be added to each target to automate the whole process. Figure 8 shows the typical case of a target-based survey. Here, two synchronized Nikon D70 cameras with 20 mm Sigma lenses were employed. Several targets placed in particular positions were tracked after their semi-automatic matching for the first epoch. The method used during the tracking phase is based on cross-correlation and LSM. More details about this test are described in the next section.

However, during some analysis targets cannot be fixed, thus we implemented a solution based on the texture of the object and projective geometry. This new method is based on detectors and descriptors capable of determining tie points among the images. In our implementation we use two operators able to extract and match these image correspondences: SIFT (Scale Invariant Feature Transform) [41] and SURF (Speeded Up Robust Features) [42]. They are invariant to changes in rotation (around the optical axis of the camera) and scale, and are robust to affine deformations and changes in illumination. The method is based on the extraction of the features during the first epoch (using the detector) and the identification of the correspondences among multiple images (using the descriptor). The main advantage given by these operators is the possibility to match tie points by analyzing the descriptor, which is a vector that contains information about the local characteristics of the extracted features: tie points are matched by using the 128-element vector associated to each feature without any preliminary information. The L2 norm of the differences (Euclidean distance) is employed. More details about this procedure can be found in [43]. The choice of the matcher (SIFT or SURF) depends on the characteristics of the object. Generally SIFT finds more points than SURF, but it is computationally more expensive. On the other hand, if an object has a good texture SURF provides a good number of image points.

At the end of the matching phase several outliers can be found, especially in the case of repetitive patterns. We remove all these wrong correspondences with the robust estimation of the fundamental matrix **F** [44], which is a 3 × 3 matrix of rank 2 that encapsulates the geometry of an uncalibrated stereo pair. If the scene is planar, it is more convenient to estimate the essential matrix **E** [45], because a 2D scene is a critical case for the **F**-matrix. We use the *Least Median of Squares* (LMedS) method [46] to estimate **F** (or **E**) with 7 image points [47].

Given a set of image correspondences **x***_i_* = (*x_i_*, *y_i_*, 1)*^T^* ↔ (*x_i_′*, *y_i_′*, 1)*^T^* = **x***_i_′* between two images picturing the same object from different camera stations, the condition **x***_i_*′*^T^* **F x***_i_* = 0 must be satisfied. This condition can be easily demonstrated by considering that the **F**-matrix represents a connection between a point in the first image and the epipolar line in the second one: **l***_i_′* = **Fx***_i_*, in which points and lines are expressed by homogeneous vectors. Indeed, the dot product between a point in the second image and the epipolar lines of the corresponding point in the first one must be zero (**x***^iT^* **l***_i_* = 0) because the point lies on the line.

In this work robust techniques play a fundamental role. They allow an efficient detection of all mismatches and are mandatory in the case of fully automated techniques. Normally, these procedures are based on the selection of minimal dataset and the following estimation of several **F**-matrices with the presented methodology. However, we are not directly interested in the value of the computed **F**, but we want to detect wrong correspondences. In this case the **F**-matrix is extremely useful, because all mismatches given by the comparison between the detectors can be removed by analyzing their image coordinates, without any consideration about the 3D geometry of the object. A method based on seven corresponding points represent the minimal case for the estimation of **F**. In fact, the **F**-matrix has a scale ambiguity that coupled with the singular constraint det(**F**) = 0 reduces the number of independent elements to seven. A solution can be estimated using the following system:

(28) $$[x_{i}^{\prime }x_{i}\;\;x_{i}^{\prime }y_{i}\;\;x_{i}^{\prime }\;\;y_{i}^{\prime }x_{i}\;\;y_{i}^{\prime }y_{i}\;\;y_{i}^{\prime }\;\;x_{i}\;\;y_{i}\;\;1]\left[\begin{array}{c} f_{1}\\ ...\\ f_{9} \end{array}\right]=0$$

where *f*_1_, …, *f*_9_ are the nine elements of **F**. The solution is a 2D space of the form α**F**_1_ + (1 − α)**F**_2_ = 0, which coupled with the determinant constraint gives det|α**F**_1_ + (1 − α)**F**_2_| = 0. This last equation is a cubic polynomial equation in α that can be easily solved.

The LMedS technique evaluates each solution with the median symmetric epipolar distance to the data [48]. The solution which minimizes the median is chosen. To estimate **F**, subsets of seven correspondences are randomly extracted from the original dataset. The minimum number of trials *m_S_* to obtain an error-free subsample with a given probably *P* and an expected fraction of outliers *ɛ* is:

(29) $$m_{s}>\frac{\text{log}(1-P)}{\text{log}(1-{\left(1-ɛ\right)}^{p})}$$

(*p* is the number of correspondences, *i.e.*, the parameters to estimate −7 in this case). For any subsample *k* of image coordinates a fundamental matrix is estimated, thus the median of the squared residuals is calculated by using the whole dataset of image coordinates and the distances between their epipolar lines:

(30) $$\mu_{k}=\text{median}\;[d^{2}(x_{i}^{\prime },F_{k}\;x_{i})+d^{2}(x_{i},\;F_{k}^{T}\;x_{i}^{\prime })]$$

The method does not need a preliminary threshold to classify a point as inlier (or outlier). A robust estimation of the standard deviation can be derived from the data with the relation:

(31) $$\sigma_{0}=c\left[1+\frac{5}{n-p}\right]\sqrt{\mu_{k}}$$

where *μ_k_* is the minimal median and *c* = 1.4826. Then, a weight *w_i_* based on *σ*_0_ is determined for each correspondence and is used to detect outliers (*w_i_* = 0):

(32) $$w_{i}=\left\{ \begin{array}{l} 1\;\;\;r_{i}^{2}\leq {\left(2.5\sigma_{0}\right)}^{2}\\ \;\;\;0\;\;\;\mathrm{otherwise} \end{array}\right.$$

where $r_{i}={\left[d^{2}(x_{i}^{\prime },F\;x_{i})+d^{2}(x_{i},F^{T}\;x_{i}^{\prime })\right]}^{1/2}$.

**Figure 9.**
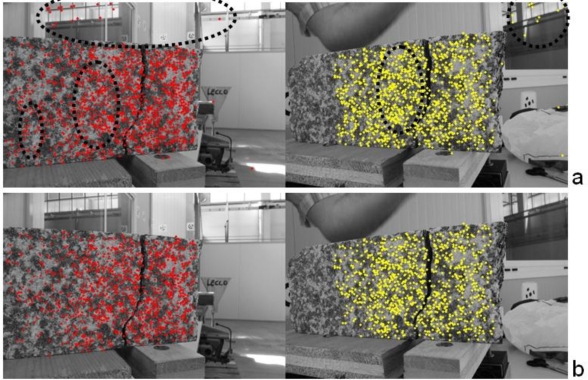
Matching results during a markerless 3D survey with two cameras: (a) point matched with the descriptors and (b) points after the robust estimation of the fundamental matrix.

After all these steps outliers can be removed and a final LSM refining is carried out to improve the precision of image coordinates. Figure 9 shows the application of the procedure. Two synchronized Nikon D80s with 20 mm lenses were placed in front of a micrometric sledge. Two pieces of rocks, which simulate a real construction element, were leant on the plates of the sledge and several shifts were given in order to compare the image measurements with the mechanical ones. The results of the matching with the descriptors are shown in Figure 9a, in which several outliers can be seen. After the robust estimation of the fundamental matrix, all these mismatches were correctly removed (Figure 9b) and can be refined *via* LSM by fixing each point in the first image and searching the homologous in the second one. Lastly, a free-net bundle adjustment was carried out and the scale of the reconstruction was fixed with a small calibrated bar. Finally, a tracking process based on cross-correlation and LSM is carried out along the image sequence and 3D points are estimated with a simple intersection by using the computed orientation parameters.

If *N* > 2 cameras are used, the images of the first epoch can be divided into (*N*^2^ − *N*)/2 pairs and the procedure is repeated for every combination. The final LSM refining is carried out by fixing the coordinates of a feature in an image (reference), while for the remaining ones the points can be found with the traditional procedure based on cross-correlation and LSM.

### 4.3. Accuracy of the Method

To check the accuracy of the implemented tool a comparison with external sensors was carried out. In this section the results related to the analysis of the examples proposed in Figures 8 and 9 are shown.

Figure 8 shows the test on a soft rock placed in a metal cylinder. A press generates a load by means of a small metal plate which simulates a foundation. The goal of this study is the determination of the characteristics of the soft rock. The image-based measurements were carried out with two Nikon D70 cameras, equipped with calibrated 20 mm lenses. Cameras were placed on tripods in order to fix their exterior orientation parameters. A solution based on targets was used. During this test two LVDTs were employed, but they provided information along the vertical line only. In this test, LVDTs were used to check the accuracy of the image-based measurements.

**Figure 10.**
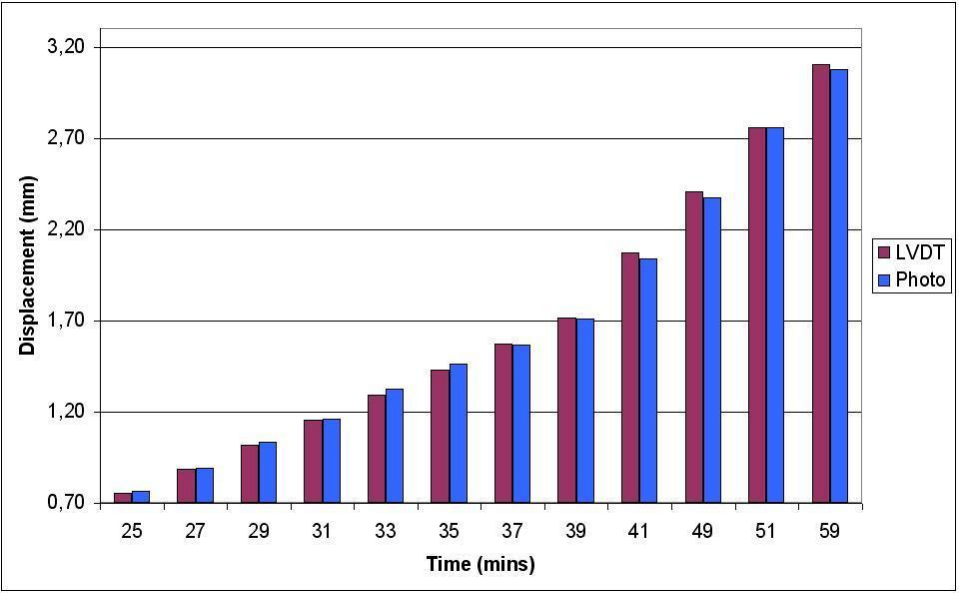
Comparison between image-based and LVDT displacements for the target-based test.

**Figure 11.**
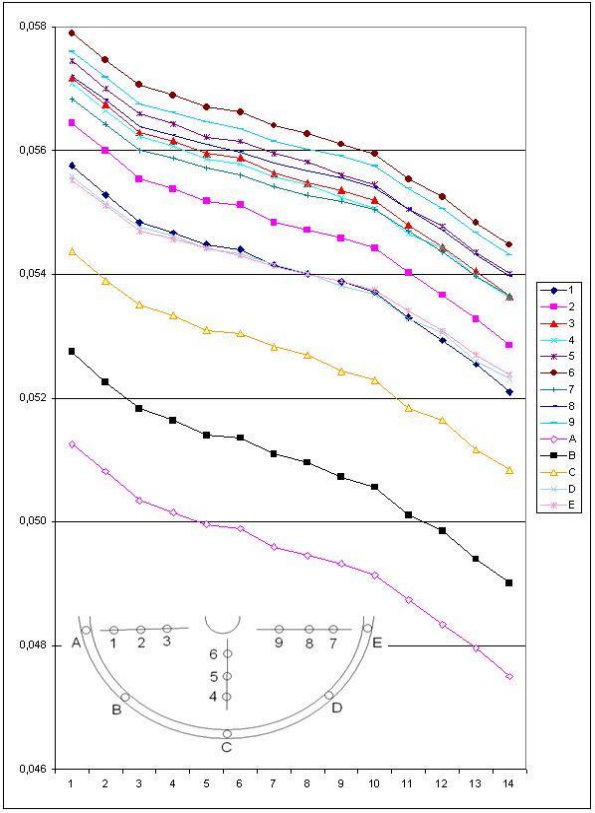
Vertical displacements (at different epochs) measured with the image-based method.

Figure 10 shows the displacements measured with a LVDT and with the image-based method, in which the vertical movements of a target close to the LVDT were employed. The standard deviation of the differences is equal to ±0.02 mm and the final displacement is larger than 3 mm. This means that the accuracy of the photogrammetric method is more than sufficient for this experiment. Moreover, the analysis of several targets distributed on the object allow the estimation of 3D movements (Figure 11) and the rotation of the foundation.

A preliminary analysis of the markerless method was carried out for the example proposed in Figure 9. A small piece of rock was placed on the fixed plate of the sledge, while a second piece on the movable part. The imaged area is 15 × 15 cm roughly. With this test some changes in aperture and depth were simulated and their magnitude was measured by using two orthogonal comparators. The results after this check demonstrated an accuracy of ±10 μm in the estimation of the aperture and ±7 μm along the depth.

**Figure 12.**
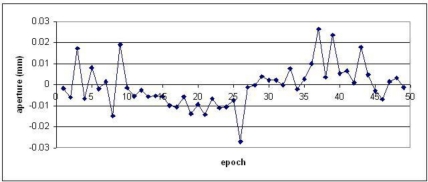

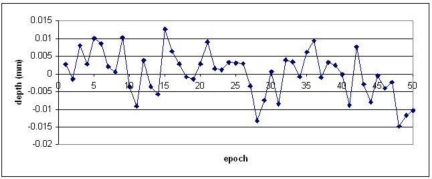
Comparison between mechanical and image-based measurements for a target-less test.

Figure 12 shows the differences between mechanical and photogrammetric measurements. Probably, the better result along the depth is due to the use of highly convergence cameras and an easier tracking phase (feature positions do not significantly change in the image). This aspect needs further investigations. However, these results are good and confirm the potentialities of the developed and implemented strategies.

## 5. Conclusions

In this paper three image-based tools for measurement of deformations in laboratory testing on construction materials were presented. Because of their user-friendliness they can be used by people who are not necessarily skilled in image analysis, computer vision, photogrammetry and vision metrology. These methods provide more information than that obtainable with traditional sensors. In addition, when targets cannot be applied the natural surface of the object can be used. In the case of bodies with a bad texture a synthetic texture can be created by painting the object. However, some new techniques based on a preliminary image enhancement of the local radiometric content of the image and feature extraction and matching can be applied to extract a sufficient number of points to complete the analysis.

The implemented image-based methods can provide 2D and 3D measurement for a vast number of points and allows the analysis of the whole body. Moreover, the procedure is highly automated and only few semi-automatic measurements for the image(s) of the first epoch are needed (e.g., target localization, visual check, removal of the scale ambiguity,…). The dynamic analysis can be considered a fully automatic phase and 2D or 3D measurements can be rapidly estimated after the end of the test. All these tools were implemented to work with building materials, in which the global deformation is limited with respect to the object size. During several standard tests on construction elements (e.g., pillars, beams…) the methods demonstrated good results even in the case of strong deformities of the body. These experimental tests demonstrated an accuracy of these methods similar to that achievable with traditional electrical or mechanical sensors, however, the use of digital cameras allows the elaboration of a larger number of 2D or 3D points with a better spatial distribution.
